# What Are the Practical Applications of Single-Cell Proteomics?

**DOI:** 10.3390/proteomes14020026

**Published:** 2026-05-18

**Authors:** Benjamin C. Orsburn

**Affiliations:** Organ Pathobiology and Therapeutics Institute, The University of Pittsburgh, Pittsburgh, PA 15206, USA; orsburn@pitt.edu

**Keywords:** single-cell proteomics, single-cell technologies, single-cell transcriptomics, low input diagnostics, spatial proteomics

## Abstract

Single-cell proteomics (SCP) is an exciting new field of study with developments in the areas of sample preparation, instrumentation and informatics. SCP has captured the imagination of biologists and clinicians and the critical interest of both academic and commercial mass-spectrometry groups. Currently (i.e., at the time this manuscript was written), SCP is still difficult and slow relative to competing single-cell technologies. What SCP may lose in relative throughput, it trades for direct analysis of protein and proteoforms, albeit with biases toward those of the highest relative concentration in each cell. These strengths may not make SCP the technology of choice for every study. This perspective is intended to identify current and future biological or clinical areas where SCP has or could have the greatest potential to advance human health and knowledge. I will also discuss applications where SCP would be less impactful than other technologies and where SCP, when mature, could play a true role in clinical diagnostics.

## 1. Introduction

Single-cell proteomics (SCP) is today’s moonshot program for mass spectrometry (MS) [1,2]. It is difficult to decide when to stop adding to the growing list of advances developed for SCP, which are trickling their way down to other aspects of liquid chromatography mass spectrometry (LCMS). While these have been more thoroughly and completely described by others [3,4], the list that has developed in just a few short years is impressive enough to note. At the bench, we have new ways to prepare samples with far less sample loss than ever before [5,6]. Striving for efficiency has led to the adoption of nanovolume sample-handling devices in many labs [7,8]. New surfactants such as n-Dodecyl-β-D-maltoside are now almost ubiquitously used in LCMS proteomics workflows, even when samples may not be accurately characterized as “ultra-low input” [9,10,11]. More rapid digestion methods are likely already in development due to increasing competition for proteomics throughput [12], but new enzymes with relatively high costs may be easier to justify when a single aliquot is sufficient for digesting thousands of single-cell or low-input samples [13]. Liquid chromatography (LC) systems are now stably flowing at lower rates than ever before, an affordance enabled by smaller commercially available nanoflow columns [5,6]. These new separation methods have even started to cut into the historic gaps in flow rates between LC and capillary electrophoresis (CE-MS) applications [14,15,16]. Cleverly leveraging traditional labeling reagents popularized by the SCoPE-MS workflows have been used for “BOOSTing” peptide signals in phosphoproteomics [17,18] and in other difficult materials [19,20,21]. In the new competitive hardware environment, single-cell proteome coverage is now just as leveraged as a sales tactic as the number of proteins in cancer cell line digests. Vendors have found a market hungry for high-resolution MS detectors with ever increasing sensitivity, and nearly all have found creative new ways to provide it [22,23]. Both high-resolution and low-resolution ion mobility separations are being leveraged to provide cleaner spectra [24,25]. Counter-intuitive methods such as wide-window acquisition have proven surprisingly successful in many applications [26,27]. Finally, the rapid adoption and simplification of proteomic algorithms enabled by artificial intelligence have now allowed unprecedented depth of analysis in both new and previously acquired data [28,29,30]. All of these tools have compounded to make SCP more accessible and available to every mass spectrometrist. The remaining question is as follows: where should it be applied, as competitive solutions for single-cell analysis have existed for a long time?

## 2. Competitive Technologies

Before going further, I should at least skim over some other relevant technologies in this space.

The most relevant competition would appear to be the many variations of single-cell transcriptome sequencing (scSeq) [31,32,33,34,35]. Although historically contentious, it is now well accepted that the correlation between transcript and protein abundance is, at best, poor in eukaryotes [36,37,38,39]. scSeq excels in detecting heterogeneity in cellular transcript abundance and achieving incredible throughput [40]. Coupled technologies such as the commercial product “Total-Seq” leverage barcoded cell surface antibodies that allow both transcript abundance and the relative abundance of target proteins to be simultaneously measured [41,42]. In the protein space, antibodies have long been bound to fluorophores for flow cytometry analysis, which can allow protein abundance inference from up to twenty targets across populations of millions of individual cells [43]. When more targets are required, antibodies and single-cell flow can be coupled with metals and inductively coupled plasma mass spectrometry (ICP-MS), respectively, by integrating tools on one’s own or through commercial setups such as the CyTOF platforms [44,45].

**Figure 1:**
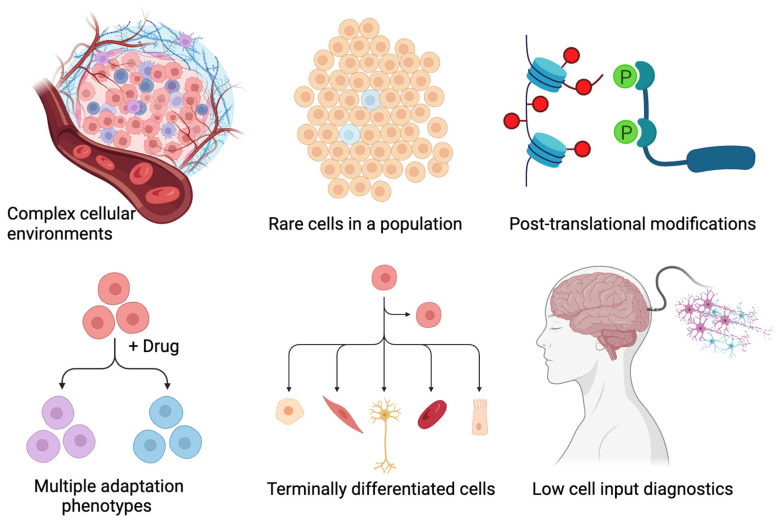
A summary of applications of single-cell proteomics that will be discussed in this *Perspective* article.

All these tools provide higher throughput but lower relative numbers of protein targets than SCP, with the obvious caveats being the challenges of utilizing antibodies in analytical workflows [46]. Of particular interest to this proteoform-centric journal, antibody-based tools are often employed against peptides or linearized versions of protein sequences. While molecular techniques such as Western blots can provide insight into the presence of alternative proteoforms and off-target binding with measurements in the mass domain, they are not readily available in these single-cell technologies and therefore provide no proteoform-level information [47,48,49]. In contrast, modern untargeted SCP can provide quantification of hundreds of proteins at a rate of hundreds of cells per day or thousands of proteins at a rate of dozens of cells per day [50]. While not all proteoform information is captured by today’s SCP techniques, advances are being made in both the post-translational modifications PTM and intact proteoform space, particularly for large types of cells [51]. It is worth noting that the field of spatial proteomics continues to develop in parallel with SCP, largely by taking advantage of the increased sensitivity of MS and LCMS hardware [52]. Today, multiple groups have leveraged laser capture microdissection to isolate areas of tissue down to the size of a single human cell [53,54,55]. While the number of ways that spatial proteomics can be performed exceeds the plans for this manuscript, I will attempt to note where they synergize. A similar and highly related concept is “single-cell type proteomics” (SC-TP), in which a population of cells with the highest possible homology are isolated and analyzed. As with spatial proteomics, SC-TP takes advantage of the fact that today’s hardware can provide near-complete proteome coverage from less than one hundred cells, with limitations that will be described within this work [22,56,57]. Ultimately, the goal of this perspective is to point out where SCP can play a role and what advantages, if any, it has over relevant competing solutions today. I’ve attempted to summarize these in the cartoon in Figure 1 and will further elaborate on each on in the following sections.

## 3. Potential Applications of Single-Cell Proteomics (SCP)

### 3.1. Complex Cellular Environments

It is likely not imprudent to wonder which cellular environments are not complex, but it is worth pointing out that clonal populations of cells are at an evolutionary disadvantage in every system on Earth [58,59]. Even cellular systems once thought to have a singular function have proven to have both single-cell and spatial heterogeneity. An example pertinent to my current work is the human liver, which is notable in that 80–90% of all cells within it are of a single cell type, the hepatocyte [60]. Primary and immortalized hepatocytes are the workhorses of the pharmaceutical industry, allowing the rapid identification of toxins and production of secondary metabolites for study prior to the use of more complex in vivo models [61,62,63]. Recent work involving spatial transcriptomics and “deep visual proteomics” or high-resolution laser capture microdissection coupled with proteomics has revealed the complexity of the intracellular environment of the simple human liver [52,64,65,66]. We now know that hepatocytes exist in tightly controlled spatial compartments, called zones [67]. While visually the same cell type, there are unique expression patterns within each zone that complicate any analysis based on cellular homogenates. Surprisingly, even in human tumors, which often arise when a single cell becomes an out-of-control dividing clonal population, other cell types are nearly always present. Without the development of vasculature to feed hungry cells within the growing tumor, the total tumor mass is strictly limited [68,69]. Therefore, homogenates of even small tumor biopsies are likely to identify proteins that are not expressed by the cancer cells themselves [70]. Dissociated cells studied via SCP can provide insight into which cells are present and in what numbers. It should be noted, however, that when cell-type markers are available, the closely related “single-cell type proteomics” (SC-TP) may provide equally useful information. SC-TP leverages the analysis of smaller numbers of cells than traditional proteomics by enriching or purifying the cells of interest with tools such as flow cytometry [71,72]. It should be noted that in the excitement for the new field of SCP, there has been some confusion regarding it and the much older field of SC-TP. This is one of many reasons for the development of a recent community-generated set of guidelines that calls for clarifying nomenclature [73]. SC-TP does, however, require ways to enrich or purify the cells of interest, which may require discoveries based on spatial proteomics or SCP to be created, as discussed further in Section 3.6.

### 3.2. Multiple Adaptation Phenotypes

It is now generally accepted that even a clonal population of cells will respond in a heterogenous manner when treated with the same drug. Many of these differences may simply result from the cell’s relative status and stage in the cell cycle [74]. In addition, the relative amount of cell-to-cell contact in each individual can lead to large changes in phenotypes. The latter example can be observed in the extreme differences in drug sensitivity that can arise when cells are grown in two-dimensional as opposed to three-dimensional culture conditions [75,76]. Many scientists are in the translational business of killing cancerous or virus-filled cells with chemotherapeutics or anti-viral therapies. Heterogeneity in response to drugs can lead to poor effectiveness or ultimately outright resistance [77,78,79,80,81]. SCP can provide new insights into these cellular responses by both identifying phenotypic responses and providing estimates of cells with each expression profile. As noted in the next section, however, rarer cells in the overall population may be easier to identify using methods with higher throughput than SCP. Conversely, however, scSeq may not provide an accurate representation of multiple responses, as nearly all drugs target active proteoforms and not all result in transcript-level responses [82,83].

### 3.3. Rare Cell Types in a Population

The simplest example of the importance of rare cell types is also likely cancer, where recurrence or metastasis is thought to be possible even if only a few cells survive a chemotherapy regimen [84,85,86]. SCP can help identify rare cells, but it should definitely be noted that this task is a challenge with today’s current throughput. If we define a rare cell type as one occurring in 4% or less of all cells analyzed, it is possible that only one of these cells (or less) will be analyzed using label-free SCP each calendar day [87]. Without repeatedly identifying these rare cells, it is possible, and even likely, that these cells will be discarded as outliers [88]. Even in multiplexed SCP, where throughput can be in the hundreds of cells per day, the reliance on a “carrier” channel of pooled cell lysates may mean that the rare cells are only spuriously detected, particularly if the proteins that make that cell special are not well-represented in the pooled carrier channel [89,90,91,92]. It is likely that higher-throughput methods of global cellular characterization such as scSeq will be more capable of identifying the presence of rare cell types. These could then be evaluated with enrichment or more targeted proteomic methods to validate these findings.

### 3.4. Terminally Differentiated Cells

Many cells either divide slowly or not at all, which limits the power of any genetics-oriented technology [59]. Cardiomyocytes and neurons are both examples of cell types that are genetically static due to their extremely low rates of cellular division. As such, heterogeneity in the proteomes of these cells is almost exclusively identified through proteoform-level differences between individual cells [93]. This is further supported by the observation that mRNA–protein correlations in the brain and heart are among the poorest in all known tissues [39]. However, the most obvious example is red blood cells, which lack a nucleus entirely and are therefore a poor option for nucleotide-sequencing techniques [94]. Pathologies obviously occur in these cell types, and SCP may be the only option for assessing heterogeneity in global expression profiles in single cells [95]. It should be noted that multiple studies involving SCP have raised the question of what actually defines a cell type. Work on macrophage differentiation [20], the dissection of cells from a mature heart [93], and the purification of cells from human blood [26,96] has suggested the existence of new cell types or sub-types that express proteome profiles that fit somewhere between those of accepted cell types.

### 3.5. Protein Post-Translational Modifications

Today, SCP is the only technology that can globally assess protein and proteoform post-translational modifications (PTMs) in single human cells [97]. There are a number of limitations to this approach, however, including the current lack of effective enrichment strategies for single cell levels of a material. While there has been progress in this area thanks to robotics and microreactors, the corresponding efforts are arguably method development projects and years from increasing PTM numbers over global analysis methods [98]. Fortunately, the recent increases in the relative speed of high-resolution LCMS systems now allow thousands of high-abundance PTMs to be detected in global proteomics data [30,99,100]. The remaining challenges are often informatic ones. Classical searches require the consideration of each peptide in both unmodified and modified forms, which can combinatorially expand search spaces. While this can be computationally challenging, a more pressing issue may be the expansion in the decoy space, which can lead to limited power in false-discovery discrimination. Modern search tools have found ways around these issues, but it is easier to simply search for the unmodified peptide sequences, despite the fact PTMs can be found, quantified and validated [30,99,100]. By simply looking for likely PTMs, early work in this SCP field identified eight different classes of PTMs in high-abundance proteins in individual human cancer cells, even at a coverage level that is low by today’s standards [97]. Further work by my group [81,101] and others [102,103] has demonstrated the robust nature of PTM identification for high-abundance proteins. As the depth of SCP data continues to increase, the number of PTMs identified in each cell will certainly continue to increase too (as reviewed in [104]). One notable limitation today is the relative difficulty of identifying PTMs in data-independent analysis (DIA) LCMS data. While the peptide PTM ions and fragments are likely well represented in these spectra, multiple challenges still exist. The first is simply the increased relative complexity of each tandem mass spectrum. Few chemical bonds behave as predictably during fragmentation as peptide bonds, and PTM fragmentation complicates spectra in a number of ways [105]. A second challenge is the increased reliance of LCMS scientists on using deep learning algorithms to predict peptide fragmentation patterns. While multiple tools that can extend deep learning to peptide PTMs have been described, they are largely experimental and poorly adopted and have been exclusively demonstrated functionality on Orbitrap hardware [106]. Recent work has, however, demonstrated the identification of both PTMs and mutations leading to single-amino-acid variants in single-cell DIA data. This approach leverages classically generated libraries from cellular homogenates analyzed with data-dependent methods. If nothing else, this study serves as a powerful proof of concept that single-cell PTMs will later be found in reanalysis with more sophisticated algorithms or improved libraries [102,107].

### 3.6. Identification of New Cell-Type Markers for Research and Pathology Applications

**Figure 2:**
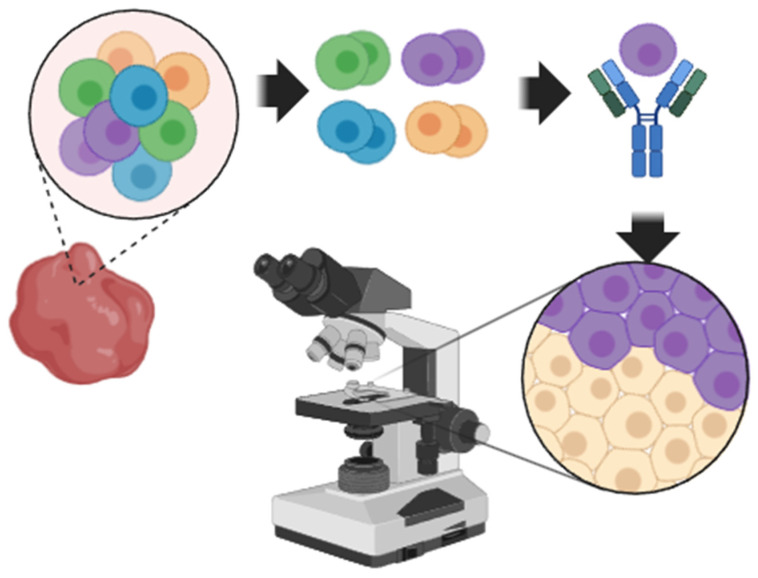
A cartoon describing the dissociation of cells for SCP analysis for the identification of cell markers and eventual antibodies for use in microscopy-based pathology assays.

As mentioned in previous sections, antibodies can be leveraged for both homogenate, single-cell, and single-cell-type analyses. These tasks all depend on the confident identification of cell-type markers for antibodies to be raised against. One direct application of SCP currently under exploration is the identification of new cell-type markers. Deep visual proteomics with single-cell resolution has been successfully applied to multiple models, and new antibodies for classic-microscopy-based pathology assays and other uses are already in development [52,54,108,109]. While these discoveries currently rely on spatial proteomics, it is not a stretch to imagine how SCP can serve in such a manner. Due to the biases of SCP toward the proteins with the highest relative concentrations, protein targets identified should prove particularly amenable to antibody-based detection [110,111]. Figure 2 is a cartoon that illustrates this workflow. A caveat for this approach is that LCMS-based proteomics has historically struggled with cell surface and membrane proteins, which are often used as cell-type markers. These limitations are largely due to the lack of basic residues such as lysine and arginine in membrane proteins and the dependence of LCMS proteomics on enzymes that preferentially cut at these residues [112,113]. With the recent emergence of low-cost, high-specificity enzymes from archaea, this may soon be a limitation of the past [114]. The advantage that SCP has over scSeq in the identification of new pathological markers is almost entirely due to the low or non-existent concordance between transcript and protein abundance, which has recently been demonstrated to exist even at the level of single cells analyzed simultaneously with both techniques [7,38].

### 3.7. Low-Cell-Input Diagnostics

Perhaps the most exciting avenue for SCP in the future is the promise of minimally invasive diagnostics that harvest small numbers of cells for analysis. For example, we have long known that when pancreatic cancer is caught early, it is far more likely to be treatable than when it is detected at later stages [115]. The pancreas, like many organs, is simply difficult to sample. Multiple studies have demonstrated success in microsampling or micro-biopsies in tissues for which larger sampling methods are too dangerous or detrimental to the patients to be applied [116]. In one study, up to seven thousand cells were extracted from the pancreases of patients with these methods, although the average number of cells was far lower throughout the patient population studied. While single cells can be extracted from a patient, limited diagnostic efficacy has been reported from such approaches when scSeq is applied to these isolated cells. As powerful as scSeq is, it does not excel in the analysis of small numbers of cells, as the low signal-to-noise ratio of these data is improved through the acquisition of thousands of repeated single-cell measurements. Without thousands of samples of each cell type, discriminatory power is too low for diagnostic development [7,20]. In addition, the primary mutations in pancreatic cancer can be single mutations, which lead to overactivation of signaling cascades, which are primarily phosphorylation and intrinsically invisible in transcript-level measurements [78,117,118]. While antibody-based techniques can allow direct identification of these sites in single cells, the small number of potential targets in these assays can propagate false-negative findings [119,120]. While we have yet to see the application of low-input or single cells in living-patient diagnostics, examples in single-cell spatial proteomics of patient slides have demonstrated the closest thing [52,53,54]. If you would like me to cancel events in my calendar and lie to students about where I am, please tell me that you would like to discuss applying SCP to diagnostic development.

## 4. Conclusions, Challenges and Future Directions

It is increasingly difficult to keep up with the advances of single-cell proteomics, even for researchers such as this author, who has labs solely dedicated to this field of research. For example, during the peer-review process for this perspective, multiple studies highlighted the value of applying SCP to understanding human fertility and early development [51,121,122]. While there are undoubtedly further applications to consider, the barrier for entry into the field is still remarkably high. Few proteomics labs have the hardware and skills required for isolating single cells. While hardware can be purchased [13], maintaining cells for meaningful biological studies requires skill and experience [88,123]. If these skills are present or can be found through collaboration, the challenges of data analysis and interpretation lurk at the end for new practitioners. While attempts are being made to address the informatics challenges, they should be a clear priority for the field in order for it to move forward [26,88,89,124,125,126,127,128,129]. While this perspective will likely be out of date by the time you are reading it, I hope it can provide some insight into when SCP might make a difference in biological and clinical discovery.

## Data Availability

No new data were created or analyzed in this study.

## Abbreviations

The following abbreviations are used in this manuscript:

SCP	Single-cell proteomics
SC-TP	Single-cell type proteomics
scSeq	Single-cell transcriptomics
MS	Mass spectrometry
LCMS	Liquid chromatography mass spectrometry
DIA	Data independent analysis
PTM(s)	Post-translational modification(s)

## References

[1] Slavov N. (2020). Unpicking the Proteome in Single Cells. Science.

[2] Slavov N. (2022). Scaling Up Single-Cell Proteomics. Mol. Cell. Proteom..

[3] Ctortecka C., Mechtler K. (2021). The Rise of Single-Cell Proteomics. Anal. Sci. Adv..

[4] Ahmad R., Budnik B. (2023). A Review of the Current State of Single-Cell Proteomics and Future Perspective. Anal. Bioanal. Chem..

[5] Kelly R., Zhu Y., Liang Y., Cong Y., Piehowski P., Dou M., Zhao R., Qian W.-J., Burnum-Johnson K., Ansong C. (2019). Single Cell Proteome Mapping of Tissue Heterogeneity Using Microfluidic Nanodroplet Sample Processing and Ultrasensitive LC-MS. J. Biomol. Tech..

[6] Cong Y., Motamedchaboki K., Misal S.A., Liang Y., Guise A.J., Truong T., Huguet R., Plowey E.D., Zhu Y., Lopez-Ferrer D. (2021). Ultrasensitive Single-Cell Proteomics Workflow Identifies > 1000 Protein Groups per Mammalian Cell. Chem. Sci..

[7] Fulcher J.M., Markillie L.M., Mitchell H.D., Williams S.M., Engbrecht K.M., Degnan D.J., Bramer L.M., Moore R.J., Chrisler W.B., Cantlon-Bruce J. (2024). Parallel Measurement of Transcriptomes and Proteomes from Same Single Cells Using Nanodroplet Splitting. Nat. Commun..

[8] Leduc A., Huffman R.G., Cantlon J., Khan S., Slavov N. (2022). Exploring Functional Protein Covariation across Single Cells Using NPOP. Genome Biol..

[9] Tsai C.-F., Zhang P., Scholten D., Martin K., Wang Y.-T., Zhao R., Chrisler W.B., Patel D.B., Dou M., Jia Y. (2021). Surfactant-Assisted One-Pot Sample Preparation for Label-Free Single-Cell Proteomics. Commun. Biol..

[10] Nie S., O’Brien Johnson R., Livson Y., Greer T., Zheng X., Li N. (2022). Maximizing Hydrophobic Peptide Recovery in Proteomics and Antibody Development Using a Mass Spectrometry Compatible Surfactant. Anal. Biochem..

[11] Muneer G., Chen C.-S., Lee T.-T., Chen B.-Y., Chen Y.-J. (2024). A Rapid One-Pot Workflow for Sensitive Microscale Phosphoproteomics. J. Proteome Res..

[12] Sun B.B., Chiou J., Traylor M., Benner C., Hsu Y.-H., Richardson T.G., Surendran P., Mahajan A., Robins C., Vasquez-Grinnell S.G. (2023). Plasma Proteomic Associations with Genetics and Health in the UK Biobank. Nature.

[13] Sanchez-Avila X., Truong T., Xie X., Webber K.G.I., Johnston S.M., Lin H.-J.L., Axtell N.B., Puig-Sanvicens V., Kelly R.T. (2023). Easy and Accessible Workflow for Label-Free Single-Cell Proteomics. J. Am. Soc. Mass Spectrom..

[14] Bagwe K., Gould N., Johnson K.R., Ivanov A.R. (2023). Single-Cell Omic Molecular Profiling Using Capillary Electrophoresis-Mass Spectrometry. TrAC Trends Anal. Chem..

[15] Johnson K.R., Gao Y., Greguš M., Ivanov A.R. (2022). On-Capillary Cell Lysis Enables Top-down Proteomic Analysis of Single Mammalian Cells by CE-MS/MS. Anal. Chem..

[16] Johnson K.R., Greguš M., Kostas J.C., Ivanov A.R. (2022). Capillary Electrophoresis Coupled to Electrospray Ionization Tandem Mass Spectrometry for Ultra-Sensitive Proteomic Analysis of Limited Samples. Anal. Chem..

[17] Chua X.Y., Mensah T., Aballo T., Mackintosh S.G., Edmondson R.D., Salomon A.R. (2020). Tandem Mass Tag Approach Utilizing Pervanadate BOOST Channels Delivers Deeper Quantitative Characterization of the Tyrosine Phosphoproteome. Mol. Cell. Proteom..

[18] Tsai C.-F., Zhao R., Williams S.M., Moore R.J., Schultz K., Chrisler W.B., Pasa-Tolic L., Rodland K.D., Smith R.D., Shi T. (2020). An Improved Boosting to Amplify Signal with Isobaric Labeling (IBASIL) Strategy for Precise Quantitative Single-Cell Proteomics *. Mol. Cell. Proteom..

[19] Budnik B., Levy E., Harmange G., Slavov N. (2018). SCoPE-MS: Mass Spectrometry of Single Mammalian Cells Quantifies Proteome Heterogeneity during Cell Differentiation. Genome Biol..

[20] Specht H., Emmott E., Petelski A.A., Huffman R.G., Perlman D.H., Serra M., Kharchenko P., Koller A., Slavov N. (2021). Single-Cell Proteomic and Transcriptomic Analysis of Macrophage Heterogeneity Using SCoPE2. Genome Biol..

[21] Végvári Á., Rodriguez J.E., Zubarev R.A. (2022). Single-Cell Chemical Proteomics (SCCP) Interrogates the Timing and Heterogeneity of Cancer Cell Commitment to Death. Anal. Chem..

[22] Heil L.R., Damoc E., Arrey T.N., Pashkova A., Denisov E., Petzoldt J., Peterson A.C., Hsu C., Searle B.C., Shulman N. (2023). Evaluating the Performance of the Astral Mass Analyzer for Quantitative Proteomics Using Data-Independent Acquisition. J. Proteome Res..

[23] Brunner A.-D., Thielert M., Vasilopoulou C., Ammar C., Coscia F., Mund A., Hoerning O.B., Bache N., Apalategui A., Lubeck M. (2022). Ultra-High Sensitivity Mass Spectrometry Quantifies Single-Cell Proteome Changes upon Perturbation. Mol. Syst. Biol..

[24] Müller F., Birklbauer M.J., Bubis J., Stejskal K., Dorfer V., Mechtler K. (2025). Breaking Barriers in Crosslinking Mass Spectrometry with Enhanced Throughput and Sensitivity Using Orbitrap Astral. Nat. Commun..

[25] Vasilopoulou C.G., Sulek K., Brunner A.D., Meitei N.S., Schweiger-Hufnagel U., Meyer S.W., Barsch A., Mann M., Meier F. (2020). Trapped Ion Mobility Spectrometry and PASEF Enable In-Depth Lipidomics from Minimal Sample Amounts. Nat. Commun..

[26] Petrosius V., Aragon-Fernandez P., Üresin N., Kovacs G., Phlairaharn T., Furtwängler B., Op De Beeck J., Skovbakke S.L., Goletz S., Thomsen S.F. (2023). Exploration of Cell State Heterogeneity Using Single-Cell Proteomics through Sensitivity-Tailored Data-Independent Acquisition. Nat. Commun..

[27] Matzinger M., Müller E., Dürnberger G., Pichler P., Mechtler K. (2023). Robust and Easy-to-Use One-Pot Workflow for Label-Free Single-Cell Proteomics. Anal. Chem..

[28] Demichev V., Messner C.B., Vernardis S.I., Lilley K.S., Ralser M. (2020). DIA-NN: Neural Networks and Interference Correction Enable Deep Proteome Coverage in High Throughput. Nat. Methods.

[29] Yu F., Haynes S.E., Teo G.C., Avtonomov D.M., Polasky D.A., Nesvizhskii A.I. (2020). Fast Quantitative Analysis of TimsTOF PASEF Data with MSFragger and IonQuant. Mol. Cell. Proteom..

[30] Kong A.T., Leprevost F.V., Avtonomov D.M., Mellacheruvu D., Nesvizhskii A.I. (2017). MSFragger: Ultrafast and Comprehensive Peptide Identification in Mass Spectrometry-Based Proteomics. Nat. Methods.

[31] Shapiro E., Biezuner T., Linnarsson S. (2013). Single-Cell Sequencing-Based Technologies Will Revolutionize Whole-Organism Science. Nat. Rev. Genet..

[32] Hwang B., Lee J.H., Bang D. (2018). Single-Cell RNA Sequencing Technologies and Bioinformaticspipelines. Exp. Mol. Med..

[33] Ziegenhain C., Vieth B., Parekh S., Reinius B., Guillaumet-Adkins A., Smets M., Leonhardt H., Heyn H., Hellmann I., Enard W. (2017). Comparative Analysis of Single-Cell RNA Sequencing Methods. Mol. Cell.

[34] Papalexi E., Satija R. (2018). Single-Cell RNA Sequencing to Explore Immune Cell Heterogeneity. Nat. Rev. Immunol..

[35] Kuboki Y., Fischer C.G., Beleva Guthrie V., Huang W., Yu J., Chianchiano P., Hosoda W., Zhang H., Zheng L., Shao X. (2019). Single-Cell Sequencing Defines Genetic Heterogeneity in Pancreatic Cancer Precursor Lesions. J. Pathol..

[36] Prasad T.S.K., Mohanty A.K., Kumar M., Sreenivasamurthy S.K., Dey G., Nirujogi R.S., Pinto S.M., Madugundu A.K., Patil A.H., Advani J. (2017). Integrating Transcriptomic and Proteomic Data for Accurate Assembly and Annotation of Genomes. Genome Res..

[37] Ghazalpour A., Bennett B., Petyuk V.A., Orozco L., Hagopian R., Mungrue I.N., Farber C.R., Sinsheimer J., Kang H.M., Furlotte N. (2011). Comparative Analysis of Proteome and Transcriptome Variation in Mouse. PLoS Genet..

[38] Nagaraj N., Wisniewski J.R., Geiger T., Cox J., Kircher M., Kelso J., Pääbo S., Mann M. (2011). Deep Proteome and Transcriptome Mapping of a Human Cancer Cell Line. Mol. Syst. Biol..

[39] Wang D., Eraslan B., Wieland T., Hallström B., Hopf T., Zolg D.P., Zecha J., Asplund A., Li L., Meng C. (2019). A Deep Proteome and Transcriptome Abundance Atlas of 29 Healthy Human Tissues. Mol. Syst. Biol..

[40] Sumida T.S., Hafler D.A. (2022). Population Genetics Meets Single-Cell Sequencing. Science.

[41] Lakkis J., Schroeder A., Su K., Lee M.Y.Y., Bashore A.C., Reilly M.P., Li M. (2022). A Multi-Use Deep Learning Method for CITE-Seq and Single-Cell RNA-Seq Data Integration with Cell Surface Protein Prediction and Imputation. Nat. Mach. Intell..

[42] Gayoso A., Steier Z., Lopez R., Regier J., Nazor K.L., Streets A., Yosef N. (2021). Joint Probabilistic Modeling of Single-Cell Multi-Omic Data with TotalVI. Nat. Methods.

[43] Berendzen K.W., Grefen C., Sakamoto T., Slane D., Kaufmann K., Vandepoele K. (2023). Analysis of Chromatin Accessibility, Histone Modifications, and Transcriptional States in Specific Cell Types Using Flow Cytometry. Plant Gene Regulatory Networks: Methods and Protocols.

[44] Löhr K., Borovinskaya O., Tourniaire G., Panne U., Jakubowski N. (2019). Arraying of Single Cells for Quantitative High Throughput Laser Ablation ICP-TOF-MS. Anal. Chem..

[45] Sun Q.-X., Wei X., Zhang S.-Q., Chen M.-L., Yang T., Wang J.-H. (2019). Single Cell Analysis for Elucidating Cellular Uptake and Transport of Cobalt Curcumin Complex with Detection by Time-Resolved ICPMS. Anal. Chim. Acta.

[46] Baker M. (2015). Reproducibility Crisis: Blame It on the Antibodies. Nature.

[47] Aebersold R., Agar J.N., Amster I.J., Baker M.S., Bertozzi C.R., Boja E.S., Costello C.E., Cravatt B.F., Fenselau C., Garcia B.A. (2018). How Many Human Proteoforms Are There?. Nat. Chem. Biol..

[48] Smith L.M., Kelleher N.L. (2018). Proteoforms as the next Proteomics Currency. Science.

[49] Su P., Hollas M.A.R., Butun F.A., Kanchustambham V.L., Rubakhin S., Ramani N., Greer J.B., Early B.P., Fellers R.T., Caldwell M.A. (2024). Single Cell Analysis of Proteoforms. J. Proteome Res..

[50] Smythers A., Orsburn B. (2026). The Current Economics and Throughput of Single Cell Proteomics by Liquid Chromatography Mass Spectrometry. ChemRxiv.

[51] Fisher N.P., Kanchustambham V.L., Tsui E.L., Lock C., Xu T., McDowell H.B., Pla I., Saunders D.C., Kafader J.O., Laronda M.M. (2026). Probing Proteoform Heterogeneity from Single Human Oocytes. Mol. Cell. Proteom..

[52] Mund A., Coscia F., Kriston A., Hollandi R., Kovács F., Brunner A.-D., Migh E., Schweizer L., Santos A., Bzorek M. (2022). Deep Visual Proteomics Defines Single-Cell Identity and Heterogeneity. Nat. Biotechnol..

[53] Guise A.J., Misal S.A., Carson R., Chu J.-H., Boekweg H., Van Der Watt D., Welsh N.C., Truong T., Liang Y., Xu S. (2024). TDP-43-Stratified Single-Cell Proteomics of Postmortem Human Spinal Motor Neurons Reveals Protein Dynamics in Amyotrophic Lateral Sclerosis. Cell Rep..

[54] Rosenberger F.A., Mädler S.C., Thorhauge K.H., Steigerwald S., Fromme M., Lebedev M., Weiss C.A.M., Oeller M., Wahle M., Metousis A. (2025). Deep Visual Proteomics Maps Proteotoxicity in a Genetic Liver Disease. Nature.

[55] Chakrabarti S., Makhmut A., Mohammadi A., Luo W., Wang L., Lewin G.R., Coscia F. (2026). Deep Visual Proteomics Uncovers Nociceptor Diversity and Pain Targets. Nat. Commun..

[56] Hendricks N.G., Bhosale S.D., Keoseyan A.J., Ortiz J., Stotland A., Seyedmohammad S., Nguyen C.D.L., Bui J.T., Moradian A., Mockus S.M. (2024). An Inflection Point in High-Throughput Proteomics with Orbitrap Astral: Analysis of Biofluids, Cells, and Tissues. J. Proteome Res..

[57] Stejskal K., Op de Beeck J., Dürnberger G., Jacobs P., Mechtler K. (2021). Ultra-Sensitive NanoLC-MS Using Second Generation Micro Pillar Array LC Technology with Orbitrap Exploris 480 and FAIMS PRO to Enable Single Cell Proteomics. bioRxiv.

[58] Lanz M.C., Fuentes Valenzuela L., Elias J.E., Skotheim J.M. (2023). Cell Size Contributes to Single-Cell Proteome Variation. J. Proteome Res..

[59] Hatton I.A., Galbraith E.D., Merleau N.S.C., Miettinen T.P., Smith B.M., Shander J.A. (2023). The Human Cell Count and Size Distribution. Proc. Natl. Acad. Sci. USA.

[60] Niu L., Geyer P.E., Gupta R., Santos A., Meier F., Doll S., Wewer Albrechtsen N.J., Klein S., Ortiz C., Uschner F.E. (2022). Dynamic Human Liver Proteome Atlas Reveals Functional Insights into Disease Pathways. Mol. Syst. Biol..

[61] Wiśniewski J.R., Vildhede A., Norén A., Artursson P. (2016). In-Depth Quantitative Analysis and Comparison of the Human Hepatocyte and Hepatoma Cell Line HepG2 Proteomes. J. Proteom..

[62] Soars M.G., McGinnity D.F., Grime K., Riley R.J. (2007). The Pivotal Role of Hepatocytes in Drug Discovery. Chem. Biol. Interact..

[63] Du Y., Wang J., Jia J., Song N., Xiang C., Xu J., Hou Z., Su X., Liu B., Jiang T. (2014). Human Hepatocytes with Drug Metabolic Function Induced from Fibroblasts by Lineage Reprogramming. Cell Stem Cell.

[64] Hu S., Liu S., Bian Y., Poddar M., Singh S., Cao C., McGaughey J., Bell A., Blazer L.L., Adams J.J. (2022). Single-Cell Spatial Transcriptomics Reveals a Dynamic Control of Metabolic Zonation and Liver Regeneration by Endothelial Cell Wnt2 and Wnt9b. Cell Rep. Med..

[65] Hildebrandt F., Andersson A., Saarenpää S., Larsson L., Van Hul N., Kanatani S., Masek J., Ellis E., Barragan A., Mollbrink A. (2021). Spatial Transcriptomics to Define Transcriptional Patterns of Zonation and Structural Components in the Mouse Liver. Nat. Commun..

[66] Ben-Moshe S., Itzkovitz S. (2019). Spatial Heterogeneity in the Mammalian Liver. Nat. Rev. Gastroenterol. Hepatol..

[67] Yasaka T.M., Kim C.K., Meadows V., Monga S.P. (2026). Zonation, Zonation, Zonation: The Real Estate of the Liver. Annu. Rev. Pathol. Mech. Dis..

[68] Johann D.J., Rodriguez-Canales J., Mukherjee S., Prieto D.A., Hanson J.C., Emmert-Buck M., Blonder J. (2009). Approaching Solid Tumor Heterogeneity on a Cellular Basis by Tissue Proteomics Using Laser Capture Microdissection and Biological Mass Spectrometry. J. Proteome Res..

[69] Stockwin L.H., Blonder J., Bumke M.A., Lucas D.A., Chan K.C., Conrads T.P., Issaq H.J., Veenstra T.D., Newton D.L., Rybak S.M. (2006). Proteomic Analysis of Plasma Membrane from Hypoxia-Adapted Malignant Melanoma. J. Proteome Res..

[70] Johann D.J., Mukherjee S., Prieto D.A., Veenstra T.D., Blonder J., Murray G.I. (2011). Profiling Solid Tumor Heterogeneity by LCM and Biological MS of Fresh-Frozen Tissue Sections. Laser Capture Microdissection: Methods and Protocols.

[71] Ding C., Li Y., Guo F., Jiang Y., Ying W., Li D., Yang D., Xia X., Liu W., Zhao Y. (2016). A Cell-Type-Resolved Liver Proteome *. Mol. Cell. Proteom..

[72] Azimifar S.B., Nagaraj N., Cox J., Mann M. (2014). Cell-Type-Resolved Quantitative Proteomics of Murine Liver. Cell Metab..

[73] Gatto L., Aebersold R., Cox J., Demichev V., Derks J., Emmott E., Franks A.M., Ivanov A.R., Kelly R.T., Khoury L. (2023). Initial Recommendations for Performing, Benchmarking and Reporting Single-Cell Proteomics Experiments. Nat. Methods.

[74] Arora M., Moser J., Hoffman T.E., Watts L.P., Min M., Musteanu M., Rong Y., Ill C.R., Nangia V., Schneider J. (2023). Rapid Adaptation to CDK2 Inhibition Exposes Intrinsic Cell-Cycle Plasticity. Cell.

[75] Whitney C.B., Beller N.C., Fries B.D., Lopez A., Hummon A.B. (2025). Longitudinal Proteomic Changes in HCT 116 Colon Cancer Spheroids During Growth. J. Proteome Res..

[76] Lopez A., Holbrook J.H., Kemper G.E., Lukowski J.K., Andrews W.T., Hummon A.B. (2024). Tracking Drugs and Lipids: Quantitative Mass Spectrometry Imaging of Liposomal Doxorubicin Delivery and Bilayer Fate in Three-Dimensional Tumor Models. Anal. Chem..

[77] Hata A.N., Shaw A.T. (2020). Resistance Looms for KRASG12C Inhibitors. Nat. Med..

[78] Xue J.Y., Zhao Y., Aronowitz J., Mai T.T., Vides A., Qeriqi B., Kim D., Li C., de Stanchina E., Mazutis L. (2020). Rapid Non-Uniform Adaptation to Conformation-Specific KRAS(G12C) Inhibition. Nature.

[79] Santana-Codina N., Chandhoke A.S., Yu Q., Małachowska B., Kuljanin M., Gikandi A., Stańczak M., Gableske S., Jedrychowski M.P., Scott D.A. (2020). Defining and Targeting Adaptations to Oncogenic KRASG12C Inhibition Using Quantitative Temporal Proteomics. Cell Rep..

[80] Kerr E.M., Gaude E., Turrell F.K., Frezza C., Martins C.P. (2016). Mutant Kras Copy Number Defines Metabolic Reprogramming and Therapeutic Susceptibilities. Nature.

[81] Orsburn B.C. (2023). Metabolomic, Proteomic, and Single-Cell Proteomic Analysis of Cancer Cells Treated with the KRASG12D Inhibitor MRTX1133. J. Proteome Res..

[82] Overington J.P., Al-Lazikani B., Hopkins A.L. (2006). How Many Drug Targets Are There?. Nat. Rev. Drug Discov..

[83] Landry Y., Gies J.-P. (2008). Drugs and Their Molecular Targets: An Updated Overview. Fundam. Clin. Pharmacol..

[84] Cosgrove N., Varešlija D., Keelan S., Elangovan A., Atkinson J.M., Cocchiglia S., Bane F.T., Singh V., Furney S., Hu C. (2022). Mapping Molecular Subtype Specific Alterations in Breast Cancer Brain Metastases Identifies Clinically Relevant Vulnerabilities. Nat. Commun..

[85] Ma Y., Shih C.-H., Cheng J., Chen H.-C., Wang L.-J., Tan Y., Zhang Y., Brown D.D., Oesterreich S., Lee A.V. (2025). High-Throughput Empirical and Virtual Screening To Discover Novel Inhibitors of Polyploid Giant Cancer Cells in Breast Cancer. Anal. Chem..

[86] Barkley D., Moncada R., Pour M., Liberman D.A., Dryg I., Werba G., Wang W., Baron M., Rao A., Xia B. (2022). Cancer Cell States Recur across Tumor Types and Form Specific Interactions with the Tumor Microenvironment. Nat. Genet..

[87] Nitz A.A., Giraldez Chavez J.H., Eliason Z.G., Payne S.H. (2025). Are We There Yet? Assessing the Readiness of Single-Cell Proteomics to Answer Biological Hypotheses. J. Proteome Res..

[88] Boekweg H., Payne S.H. (2023). Challenges and Opportunities for Single-Cell Computational Proteomics. Mol. Cell. Proteom..

[89] Vanderaa C., Gatto L. (2025). Scplainer: Using Linear Models to Understand Mass Spectrometry-Based Single-Cell Proteomics Data. Genome Biol..

[90] Cheung T.K., Lee C.Y., Bayer F.P., McCoy A., Kuster B., Rose C.M. (2021). Defining the Carrier Proteome Limit for Single-Cell Proteomics. Nat. Methods.

[91] Stopfer L.E., Conage-Pough J.E., White F.M. (2021). Quantitative Consequences of Protein Carriers in Immunopeptidomics and Tyrosine Phosphorylation MS2 Analyses. Mol. Cell. Proteom..

[92] Petelski A.A., Slavov N., Specht H. (2022). Single-Cell Proteomics Preparation for Mass Spectrometry Analysis Using Freeze-Heat Lysis and an Isobaric Carrier. J. Vis. Exp..

[93] Ai L., Binek A., Zhemkov V., Cho J.H., Haghani A., Kreimer S., Israely E., Arzt M., Chazarin B., Sundararaman N. (2025). Single Cell Proteomics Reveals Specific Cellular Subtypes in Cardiomyocytes Derived from Human IPSCs and Adult Hearts. Mol. Cell. Proteom..

[94] Garrett M.E., Foster M.W., Telen M.J., Ashley-Koch A.E. (2024). Nontargeted Plasma Proteomic Analysis of Renal Disease and Pulmonary Hypertension in Patients with Sickle Cell Disease. J. Proteome Res..

[95] Ctortecka C., Clark N.M., Boyle B., Seth A., Mani D.R., Udeshi N.D., Carr S.A. (2024). Automated Single-Cell Proteomics Providing Sufficient Proteome Depth to Study Complex Biology beyond Cell Type Classifications. bioRxiv.

[96] Furtwängler B., Üresin N., Richter S., Schuster M.B., Barmpouri D., Holze H., Wenzel A., Grønbæk K., Theilgaard-Mönch K., Theis F.J. (2025). Mapping Early Human Blood Cell Differentiation Using Single-Cell Proteomics and Transcriptomics. Science.

[97] Orsburn B.C., Yuan Y., Bumpus N.N. (2022). Insights into Protein Post-Translational Modification Landscapes of Individual Human Cells by Trapped Ion Mobility Time-of-Flight Mass Spectrometry. Nat. Commun..

[98] Wu Q., Cao L.-R., Jiang Y.-R., Shi S.-W., Guan Z.-Y., Wang Y., Wu J., Chen J.-B., Ying W.-X., Xu Q.-Q. (2025). Microamount Phosphopeptide-Enrichment-System-Based Phosphoproteomic Analysis for Small Numbers of Cells and Single Cells. Anal. Chem..

[99] Solntsev S.K., Shortreed M.R., Frey B.L., Smith L.M. (2018). Enhanced Global Post-Translational Modification Discovery with MetaMorpheus. J. Proteome Res..

[100] Prakash A., Ahmad S., Majumder S., Jenkins C., Orsburn B. (2019). Bolt: A New Age Peptide Search Engine for Comprehensive MS/MS Sequencing Through Vast Protein Databases in Minutes. J. Am. Soc. Mass Spectrom..

[101] Orsburn B.C. (2025). Simultaneous Single-Cell Proteomics and Epigenetic Analysis of Histone Deacetylase Inhibition in Human Cells. Commun. Biol..

[102] Mun D.-G., Bhat F.A., Joshi N., Sandoval L., Ding H., Jain A., Peterson J.A., Kang T., Pujari G.P., Tomlinson J.L. (2024). Diversity of Post-Translational Modifications and Cell Signaling Revealed by Single Cell and Single Organelle Mass Spectrometry. Commun. Biol..

[103] Cutler R., Corveleyn L., Ctortecka C., LiCausi F., Cantlon J., Jacome A.S.V., Deforce D., Vijg J., Dhaenens M., Papanastasiou M. (2025). Mass Spectrometry-Based Profiling of Single-Cell Histone Post-Translational Modifications to Dissect Chromatin Heterogeneity. Nat. Commun..

[104] Orsburn B.C., Vegvari A., Teppo J., Zubarev R.A. (2024). Analyzing Posttranslational Modifications in Single Cells. Mass Spectrometry Based Single Cell Proteomics.

[105] Lou R., Cao Y., Li S., Lang X., Li Y., Zhang Y., Shui W. (2023). Benchmarking Commonly Used Software Suites and Analysis Workflows for DIA Proteomics and Phosphoproteomics. Nat. Commun..

[106] Gessulat S., Schmidt T., Zolg D.P., Samaras P., Schnatbaum K., Zerweck J., Knaute T., Rechenberger J., Delanghe B., Huhmer A. (2019). Prosit: Proteome-Wide Prediction of Peptide Tandem Mass Spectra by Deep Learning. Nat. Methods.

[107] Boekweg H., Van Der Watt D., Truong T., Johnston S.M., Guise A.J., Plowey E.D., Kelly R.T., Payne S.H. (2022). Features of Peptide Fragmentation Spectra in Single-Cell Proteomics. J. Proteome Res..

[108] Kabatnik S., Post F., Drici L., Bartels A.S., Strauss M.T., Zheng X., Madsen G.I., Mund A., Rosenberger F.A., Moreira J. (2024). Spatial Characterization and Stratification of Colorectal Adenomas by Deep Visual Proteomics. iScience.

[109] Kabatnik S., Zheng X., Pappas G., Steigerwald S., Padula M.P., Mann M. (2025). Deep Visual Proteomics Reveals DNA Replication Stress as a Hallmark of Signet Ring Cell Carcinoma. npj Precis. Oncol..

[110] Orsburn B.C. (2021). Evaluation of the Sensitivity of Proteomics Methods Using the Absolute Copy Number of Proteins in a Single Cell as a Metric. Proteomes.

[111] Truong T., Webber K.G.I., Madisyn Johnston S., Boekweg H., Lindgren C.M., Liang Y., Nydegger A., Xie X., Tsang T.-M., Jayatunge D.A.D.N. (2023). Data-Dependent Acquisition with Precursor Coisolation Improves Proteome Coverage and Measurement Throughput for Label-Free Single-Cell Proteomics **. Angew. Chem. Int. Ed..

[112] Orsburn B.C., Stockwin L.H., Newton D.L. (2011). Challenges in Plasma Membrane Phosphoproteomics. Expert Rev. Proteom..

[113] Fried M., Wendler J.P., Mutabingwa T.K., Duffy P.E. (2004). Mass Spectrometric Analysis of Plasmodium Falciparum Erythrocyte Membrane Protein-1 Variants Expressed by Placental Malaria Parasites. Proteomics.

[114] Yannone S.M., Tuteja V., Goleva O., Leung D.Y.M., Stotland A., Keoseyan A.J., Hendricks N.G., Parker S., Van Eyk J.E., Kreimer S. (2025). Toward Real-Time Proteomics: Blood to Biomarker Quantitation in under One Hour. Anal. Chem..

[115] Solmi L., Muratori R., Bacchini P., Primerano A., Gandolfi L. (1992). Comparison between Echo-Guided Fine-Needle Aspiration Cytology and Microhistology in Diagnosing Pancreatic Masses. Surg. Endosc..

[116] Rogers C., Samore W., Pitman M.B., Chebib I. (2020). Solitary Fibrous Tumor Involving the Pancreas: Report of the Cytologic Features and First Report of a Primary Pancreatic Solitary Fibrous Tumor Diagnosed by Fine-Needle Aspiration Biopsy. J. Am. Soc. Cytopathol..

[117] Drosten M., Barbacid M. (2020). Targeting the MAPK Pathway in KRAS-Driven Tumors. Cancer Cell.

[118] Shackelford R.E., Whitling N.A., McNab P., Japa S., Coppola D. (2012). KRAS Testing: A Tool for the Implementation of Personalized Medicine. Genes Cancer.

[119] Ye X., Chan K.C., Waters A.M., Bess M., Harned A., Wei B.R., Loncarek J., Luke B.T., Orsburn B.C., Hollinger B.D. (2016). Comparative Proteomics of a Model MCF10A-KRasG12V Cell Line Reveals a Distinct Molecular Signature of the KRasG12V Cell Surface. Oncotarget.

[120] McAllister F., Bailey J.M., Alsina J., Nirschl C.J., Sharma R., Fan H., Rattigan Y., Roeser J.C., Lankapalli R.H., Zhang H. (2014). Oncogenic Kras Activates a Hematopoietic-to-Epithelial IL-17 Signaling Axis in Preinvasive Pancreatic Neoplasia. Cancer Cell.

[121] Wu T., Jiang L., Mukhtar T., Wang L., Jian R., Wang C., Trinh T., Kriegstein A.R., Snyder M., Li J. (2026). Single-Cell Proteomic Landscape of the Developing Human Brain. Nat. Biotechnol..

[122] Zhang H., Zhang H., Huang C., Zeng Q., Tian C., Yang J., He F., Yang Y. (2025). Deep Profiling of Oocyte Aging Enabled by Simple One-Step Vial-Based Pretreatment and Single-Cell Proteomics. JACS Au.

[123] Boekweg H., Guise A.J., Plowey E.D., Kelly R.T., Payne S.H. (2021). Calculating Sample Size Requirements for Temporal Dynamics in Single-Cell Proteomics. Mol. Cell. Proteom..

[124] Warshanna A., Orsburn B.C. (2023). SCP Viz—A Universal Graphical User Interface for Single Protein Analysis in Single Cell Proteomics Datasets. bioRxiv.

[125] Lähnemann D., Köster J., Szczurek E., McCarthy D.J., Hicks S.C., Robinson M.D., Vallejos C.A., Campbell K.R., Beerenwinkel N., Mahfouz A. (2020). Eleven Grand Challenges in Single-Cell Data Science. Genome Biol..

[126] Vanderaa C., Gatto L. (2021). Replication of Single-Cell Proteomics Data Reveals Important Computational Challenges. bioRxiv.

[127] Hicks S.C., Townes F.W., Teng M., Irizarry R.A. (2018). Missing Data and Technical Variability in Single-Cell RNA-Sequencing Experiments. Biostatistics.

[128] Kalxdorf M., Müller T., Stegle O., Krijgsveld J. (2021). IceR Improves Proteome Coverage and Data Completeness in Global and Single-Cell Proteomics. Nat. Commun..

[129] Koutrouli M., Finotello F., Schoof E.M., Roussos P., Vierdag W.-M., Diedrich L., Meyer-Bender M., Kuehl M., Nimo J., Vöhringer H. (2026). Unpaired Data as a First-Order Challenge in Single-Cell and Spatial Proteomics. Nat. Biotechnol..

